# Alteration of osteoblast arrangement via direct attack by cancer cells: New insights into bone metastasis

**DOI:** 10.1038/srep44824

**Published:** 2017-03-17

**Authors:** Yumi Kimura, Aira Matsugaki, Aiko Sekita, Takayoshi Nakano

**Affiliations:** 1 Division of Materials and Manufacturing Science, Graduate School of Engineering, Osaka University, 2-1 Yamada-oka, Suita, Osaka 565-0871, Japan

## Abstract

Intact bone tissue exhibits a characteristic anisotropic microstructure derived from collagen fiber alignment and the related *c*-axis orientation of apatite crystals, which govern the mechanical properties of bone tissue. In contrast, tumor-invaded bone exhibits a disorganized, less-aligned microstructure that results in severely disrupted mechanical function. Despite its importance both in basic principle and in therapeutic applications, the classical understanding of bone metastasis is limited to alterations in bone mass regulated by metastatic cancer cells. In this study, we demonstrate a novel mechanism underlying the disruption of bone tissue anisotropy in metastasized bone. We observed that direct attack by cancer cells on osteoblasts induces the less-organized osteoblast arrangement. Importantly, the crystallographic anisotropy of bone tissue is quantitatively determined by the level of osteoblast arrangement. Osteoblast arrangement was significantly disrupted by physical contact with cancer cells such as osteolytic melanoma B16F10, breast cancer MDA-MB-231, and osteoblastic prostate cancer MDA-PCa-2b cells. The present findings demonstrate that the abnormal arrangement of osteoblasts induced by physical contact with cancer cells facilitates the disorganized microstructure of metastasized bone.

The crosstalk between cancer cells and osteoblasts plays a key role in bone metastasis and tumor progression both in osteoblastic and osteolytic metastasis^1^,^2^. Tumor invasion and metastasis effect dynamic changes in targeted organs both histologically and genetically^3^. Interestingly, our recent study revealed that cancer cells alter the anisotropic microstructure of metastasized bone^4^,^5^. Homing of cancer cells to bone induces disrupted mechanical function derived from the disorganized microstructure associated with the less aligned crystallographic structure of apatite *c*-axis with the hexagonal lattice of the P6_3_/m space group. Tissue or organ patterning is controlled by collaborative operations of directional behaviors in constituent cells involving cellular polarity, arrangement, and migration. This process is essential for exerting tissue or organ-specific biological and/or mechanical functions. In particular, bone tissue exhibits the characteristic anisotropic architecture derived from the orientation of collagen fibers and the related apatite crystals^6^. This strongly depends on the anatomical bone portion^7^, which governs the mechanical functions of bone tissue rather than bone mass^8^,^9^. Dentin also exhibits two-dimensional alignment of collagen/apatite in conjunction with the dentinal tubules traveling, resulting in the specific three-dimensional mechanical property^10^. Osteoblast alignment is a crucial driver for construction of healthy bone with anisotropic microstructure. We have reported significant association between osteoblast arrangement and bone matrix architecture^11^,^12^,^13^. Despite its great importance in cancer treatment, cancer cell-mediated disorganization of bone matrix architecture, as a dominant trigger for pathological fracture, has not been thoroughly investigated during the long history of cancer research. Moreover, the mechanisms underlying the alteration of bone microstructure in metastasized bone are not yet understood. Here, we propose a novel mechanism, highlighting less-organized osteoblast assembly as an essential trigger for the less-aligned bone matrix structure in metastasized bone.

Cancer cells and osteoblasts dynamically regulate each other via soluble factors and physical contact. To date, only intercellular signaling communication between cancer cells and bone cells in the context of bone metabolism has been identified and recognized as a therapeutic target. For example, several osteoblast-stimulating factors such as bone morphogenetic proteins (BMPs), insulin-like growth factor-1 (IGF-1), and transforming growth factor-β (TGF-β) have been detected in prostate cancer cells^14^. They induce the upregulation of transcriptional factors that control osteogenesis. Physical contact between osteoblasts and prostate cancer cells have also been reported to upregulate the expression of specific osteoclastogenesis related genes^15^. The function of metastasized tissue is strongly dependent on the cellular organization of targeted tissues. However, the effects of cancer cells on cellular arrangement in targeted tissue have not been adequately investigated.

A number of systematic studies on *in vitro* three dimensional (3D) bone metastasis models has been carried out by Hutmacher’s group^16^,^17^,^18^,^19^,^20^. The mineralized bone matrix secreted by osteoblasts have been shown to represent a validated cell culture platform, which mimics the physiological chemical and structural composition of the bone^16^. They also revealed that the adhesion between cancer cells and bone microenvironment played a significant role in cancer invasion and colonization through the establishment of the osteoblast-produced mineralized matrix, which strongly resembled the microstructure of the bone. Furthermore, by using this biomimetic bone matrix microenvironment, integrin β1 was shown to have a pivotal role in metastatic cancer colonization^17^. Sieh *et al*. established a 3D coculture model which enabled the analysis of interactions between osteoblasts and prostate cancer cells using a polycaprolcatone-tricalcium phosphate (PCL-TCP) bone-mimetic composite scaffold^18^,^19^. Recently, we had established a bone-mimetic anisotropic culture model by controlling the orientation of the substrate collagen^11^. Further development of the culture model by combining the artificially-controlled anisotropic culture substrate with the cancer-bone coculture system is necessary to study the cellular mechanisms behind the metastasis-mediated disorganization of the anisotropic bone microstructure^4^,^5^. Here, focusing on the cellular orientation in response to the collagen molecular anisotropy, coculture of cancer cells and osteoblasts was performed on an artificially controlled oriented collagen scaffold.

In the present study, the effects of cancer cells such as osteolytic melanoma B16F10, breast cancer MDA-MB-231, and osteoblastic prostate cancer MDA-PCa-2b on osteoblast arrangement were investigated for the first time. Osteoblast alignment in response to substrate collagen orientation was analyzed using indirect and direct coculture systems (Fig. 1). Healthy osteoblasts can preferentially align along the collagen molecular orientation by organizing the cytoskeletal proteins. However, osteoblasts that communicate with cancer cells via physical contact showed significantly altered responses against substrate anisotropy. The obtained findings indicate the novel molecular interactions between cancer cells and osteoblasts, which directly inhibit normal anisotropic bone tissue organization.

## Results

### Effect of indirect interactions with cancer cells on osteoblast proliferation and differentiation

**Figure 2:**
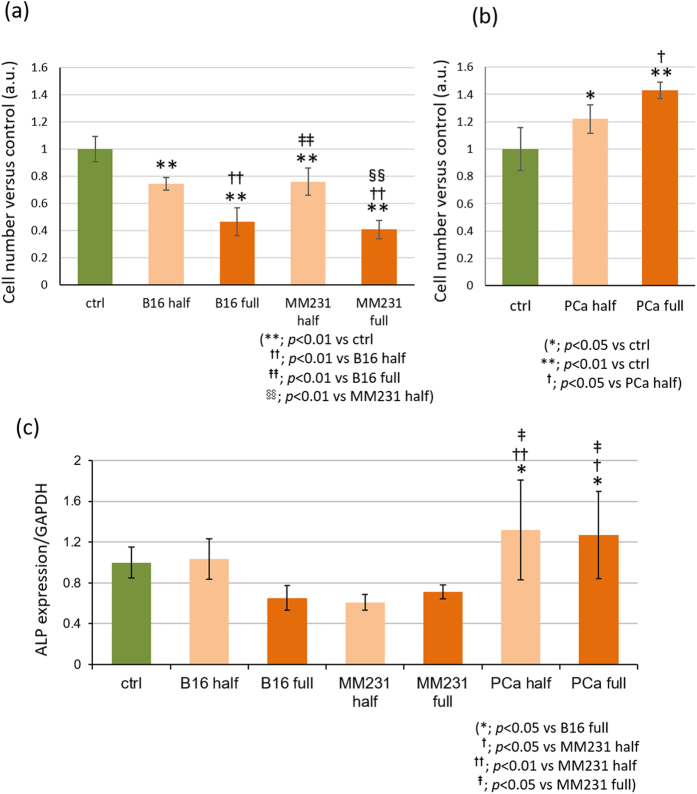
Effects of cancer cell-derived conditioned media on osteoblast proliferation and differentiation. (**a**) Osteoblast proliferation was significantly inhibited by osteolytic B16F10 and MDA-MB-231 conditioned media and (**b**) promoted by osteoblastic MDA-PCa-2b conditioned media depending on the concentration of the conditioned media. (**c**) Gene expression levels of ALP in osteoblasts cultured in osteolytic conditioned media were significantly downregulated compared to osteoblastic MDA-PCa-2b conditioned media. B16: B16F10, MM231: MDA-MB-231, PCa: MDA-PCa-2b.

Osteoblast proliferation was significantly inhibited by osteolytic B16F10- and MDA-MB-231-conditioned media (Fig. 2(a), S1). In contrast, a significant increase in cell proliferation was observed in osteoblastic MDA-PCa-2b conditioned media compared to control (Fig. 2(b)). The effect of soluble factors on osteoblast proliferation was dependent on the concentration of the conditioned media. There was no significant difference in the viability of osteoblasts in the presence of cancer cell-conditioned media compared to the control (Fig. S2).

The gene expression level of alkaline phosphatase (ALP) in osteoblasts in indirect interaction with cancer cells was analyzed by real-time RT-PCR. The expression levels of ALP in osteoblasts cultured in osteolytic conditioned media were significantly downregulated compared to that in osteoblastic MDA-PCa-2b-conditioned media (Fig. 2(c)).

### Osteoblast arrangement under indirect coculture with cancer cells

**Figure 3:**
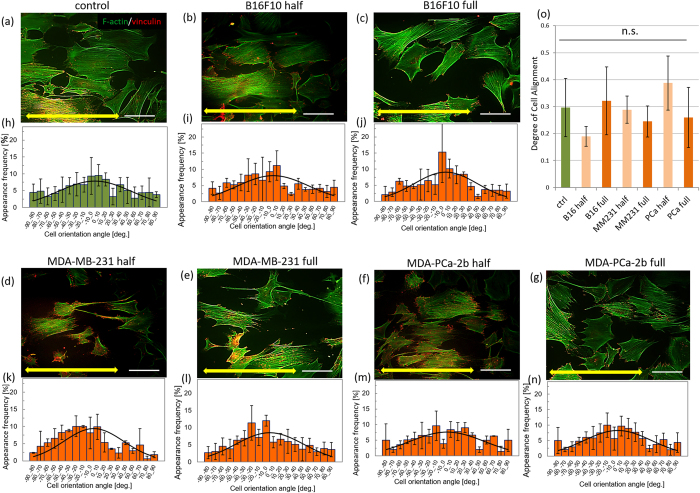
Immunocytochemical analysis of osteoblasts cultured with cancer cell-conditioned media ((**a**): control, (**b**,**d**,**f**): 50% concentration, and (**c**,**e**,**g**): 100% concentration of (**b**,**c**): mouse melanoma B16F10, (**d**,**e**): human breast cancer MDA-MB-231, (**f**,**g**): human prostate cancer MDA-PCa-2b cells conditioned media). The histograms of cell angular distribution are shown in figures (**h**–**n**). The osteoblasts are preferentially aligned along the substrate collagen orientation (yellow arrows). (**o**) The degree of cell alignment along the substrate collagen orientation. Osteoblast alignment is not significantly affected by the cancer cell-derived conditioned media. Scale bar: 100 μm.

Figure 3 shows immunofluorescence images of osteoblasts cultured with or without conditioned media on oriented collagen substrates. Osteoblasts were aligned in parallel to the substrate collagen orientation both in the control or conditioned media. Quantitative evaluation of cell orientation using fluorescent images of actin cytoskeleton and vinculin demonstrated that there is no significant difference between osteoblasts cultured in control or cancer cell-conditioned media. Figure 3 also shows the distribution of cell orientation against the substrate collagen orientation quantified by 10°. The distribution of cell alignment was concentrated to around 0° in osteoblasts cultured in control (Fig. 3(a,h)) or conditioned media (Fig. 3(b–g)). The degree of cell alignment shows no significant differences between mono-cultured osteoblasts and osteoblasts in conditioned media derived from both osteolytic and osteoblastic cancers (Fig. 3(o)).

### Intercellular adhesion between cancer cells and osteoblasts under direct coculture

**Figure 4:**
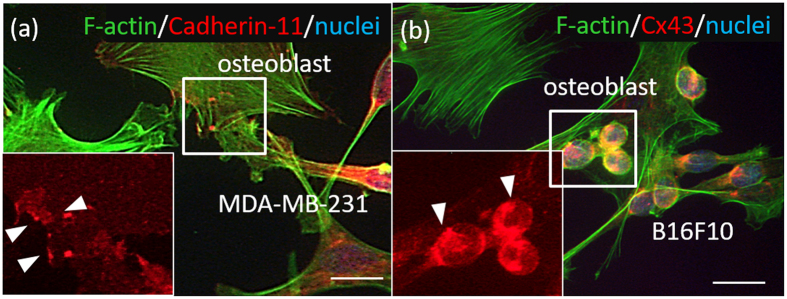
Visualization of intercellular adhesion between cancer cells and osteoblasts cultured on culture dish. (**a**) Cadherin-11 is positively expressed in the osteoblasts and MDA-MB-231. Green: F-actin, red: Cadherin-11, blue: nuclei. (**b**) Connexin 43 is expressed in the osteoblasts and B16F10. Green: F-actin, red: Cx43, blue: nuclei. Scale bar: 30 μm.

Cadherin-11 and connexin 43 were highly expressed when osteoblasts and cancer cells were cocultured (Fig. 4). Direct interactions between osteoblasts and MDA-MB-231 via cadherin-11, and between osteoblasts and B16F10 via connexin 43, were successfully visualized using immunocytochemistry.

### Osteoblast arrangement under direct coculture with cancer cells

**Figure 5:**
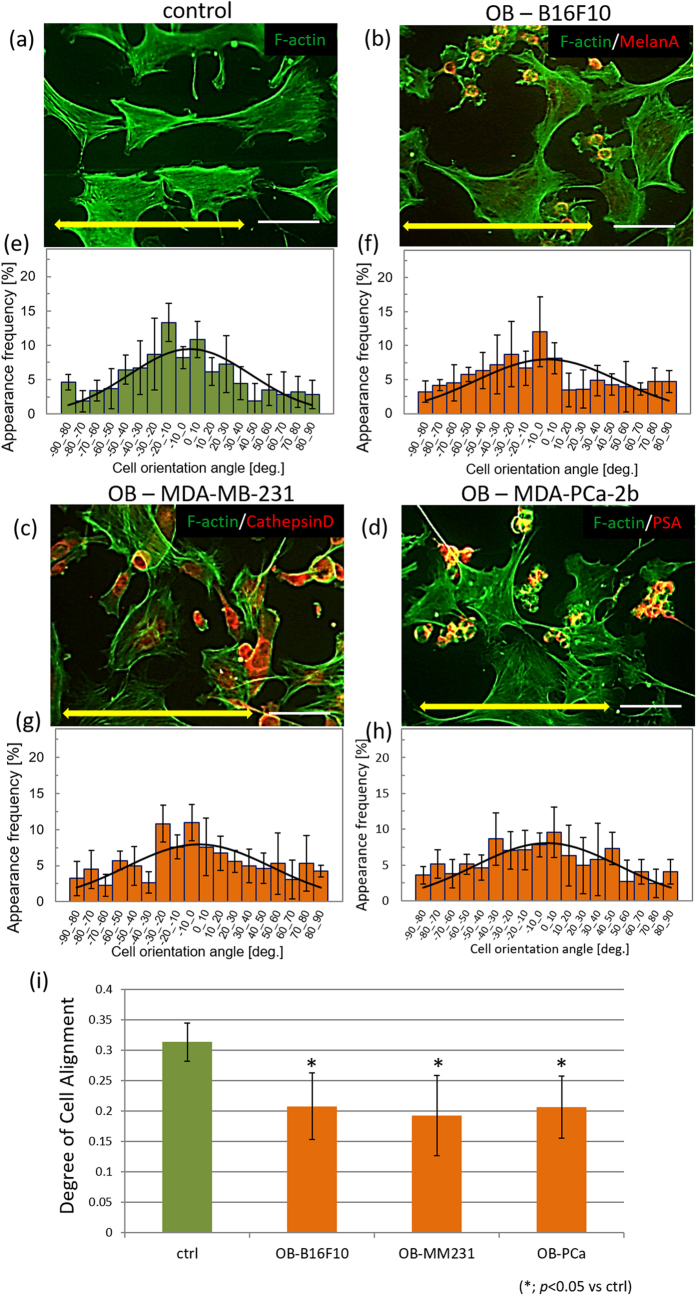
Immunocytochemical analysis of osteoblasts under direct coculture with cancer cells. (**a**) Osteoblasts align along the collagen orientation in control monoculture. (**b**,**c**,**d**) Osteoblasts cultured with cancer cells in a direct coculture system show disrupted alignment. The histograms of cell angular distribution are shown in (**e**–**h**). (**i**) Analysis of the degree of cell alignment indicates that cancer cells significantly disrupt osteoblast alignment against substrate anisotropy. Yellow arrows indicate the direction of substrate collagen orientation. OB: osteoblast. Scale bar: 100 μm.

**Figure 6:**
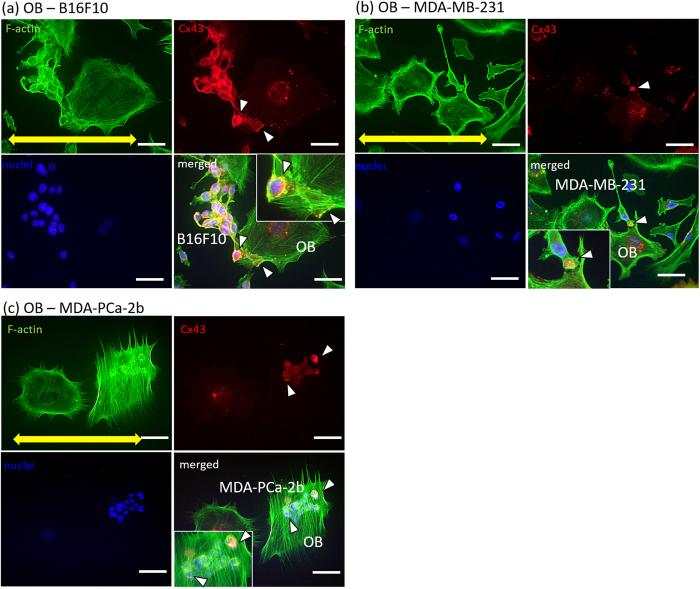
Visualization of gap junction between osteoblasts and cancer cells, including (**a**) mouse melanoma B16F10, (**b**) human breast cancer MDA-MB-231, and (**c**) human prostate cancer MDA-PCa-2b. Yellow arrows indicate the direction of substrate collagen orientation. Arrowheads indicate the Cx43-positive gap junctions between cancer cells and osteoblasts. Green: F-actin, red: Cx43, blue: nuclei. Scale bar: 50 μm. Insets show magnified images of the Cx43-positive gap junctions. Scale bar in insets: 20 μm.

The degree of substrate collagen orientation showed no difference with and without cancer cells (Fig. S5). Osteoblast alignment along the substrate collagen orientation was significantly disrupted when in contact with cancer cells. Osteoblasts were well aligned along the substrate collagen orientation in monoculture, whereas osteoblasts cocultured with cancer cells were less aligned (Fig. 5(a–h)). Quantitative evaluation of the degree of cell alignment revealed a significant decrease in alignment in osteoblasts when in contact with cancer cells (Fig. 5(i)). In particular, physical contact with three types of cancer cells via gap junctions was confirmed in less-aligned osteoblasts cultured on oriented collagen substrates (Fig. 6).

## Discussion

Cellular arrangement is crucial for the establishment of tissue or organ morphogenesis and the resulting appropriate function. Tissue organization mirrors constituent cell arrangement. In this study, we show for the first time that osteoblast arrangement, which determines bone tissue anisotropy, is regulated by cancer cells via direct intercellular communication.

The metabolic effects of cancer cells on osteoblast activity were demonstrated by focusing on cellular proliferation and differentiation behaviors under indirect coculture with cancer cells. Osteolytic and osteoblastic cancer cells play significantly identical roles in osteoblast behaviors such as cell proliferation and differentiation. Osteoblasts cultured with soluble factors derived from osteolytic B16F10 melanoma and MDA-MB-231 breast cancer cells demonstrated significantly decreased number of cells depending on the concentration of the conditioned media (Fig. 2(a), S1). However, there was no significant change in cell viability under indirect coculture with conditioned media compared to control monoculture (Fig. S2). In addition, the gene expression level of alkaline phosphatase, an index of osteoblast differentiation, was significantly decreased in osteolytic cancer cell-conditioned osteoblasts (Fig. 2(c)). Osteoblasts cultured under indirect conditions with osteoblastic MDA-PCa-2b prostate cancer cells showed significantly increased cell numbers and alkaline phosphatase expression (Fig. 2(b,c)). These results indicate that cancer cells can regulate osteoblast proliferation via soluble factors, which is consistent with results of previous studies^21^,^22^. Cancer cell-derived soluble factors that regulate osteoblast functions have been studied both *in vivo* and *in vitro*. Among them, Wnts have been shown to play a key role in bone metastasis and mediate cancer and bone cell interactions^23^. In osteoblasts, Wnt signaling pathways are known to upregulate cell proliferation^24^, and also enhance osteoblast differentiation^25^. Osteolytic cancer cells, such as breast cancer cells, overexpress Sclerostin (SOST) or Dickkopf1 (Dkk1), which play inhibiting roles in the Wnt signaling pathway, leading to downregulation of cell proliferation and differentiation. However, Endothelin-1 (ET-1) produced by prostate cancer cells inhibit Dkk-1 expression in osteoblasts^26^. Several BMPs and fibroblast growth factors (FGFs) activate Smad signaling in osteoblasts, which can accelerate osteoblast proliferation and differentiation^27^. In this study, we show that the above mentioned factors could possibly affect osteoblast behaviors involving proliferation and differentiation.

Importantly, bone matrix anisotropy is quantitatively regulated by the degree of osteoblast alignment^11^. Osteoblasts organize cytoskeletal proteins in response to scaffold anisotropy, communicate with each other, and provide oriented bone matrix depending on the cellular arrangement. The degree of cell alignment is therefore a powerful indicator of the resultant tissue organization and morphology. Apart from the metabolic effects of cancer cells on osteoblasts, cancer cell-derived soluble factors have no significant effect on osteoblast arrangement (Fig. 3). Cellular arrangement along the collagen molecular orientation is regulated via integrin-mediated interactions between the matrix interaction domain in collagen molecules and cell surface. The cancer cell-derived soluble factors are considered to have no significant effects on osteoblast-collagen interactions and the subsequent cytoskeletal rearrangement in osteoblasts.

Bone metastasis is mediated by complex interactions between tumor cells and bone- derived cells. This procedure involves direct intercellular heterotypic interactions between cancer cells and osteoblasts. Two types of intercellular interactions, cell-cell adhesion, and intercellular communication via gap junctions, were confirmed by visualization by immunocytochemistry (Fig. 4). For example, cadherin-11, a homophilic cell-cell adhesion molecule that has been implicated in cancer invasion and metastasis via direct connections with osteoblasts^28^, is expressed in MDA-MB-231 breast cancer cells and primary osteoblasts (Fig. 4(a)). Other types of cellular interactions such as gap junction communications are also reported to contribute to tumorigenesis and metastasis^29^,^30^. Connexin 43 expression in the intercellular region of melanoma cells and osteoblasts was confirmed (Fig. 4(b)). The cultivation conditions for direct coculture of cancer cells and osteoblasts were optimized by evaluating the intercellular contacts by immunocytochemical analysis, because of the difference in doubling time of cancer cells, depending on the three types of cancer cells (B16F10, MDA-MB-231 and MDA-PCa-2b) (Fig. S3).

The microenvironment of bone tissue impacts not only the establishment and progression of bone metastasis but also the reciprocal interactions between cancer cells and osteoblasts^31^. The oriented collagen substrate, which mimics the anisotropic bone matrix surface, was fabricated, and the change in cellular arrangement in response to the collagen orientation was analyzed during direct interaction with cancer cells. The degree of collagen orientation with and without cancer cells was analyzed by measuring quantitative birefringence, and it was confirmed that cancer cell-mediated alteration of substrate collagen orientation did not occur during our experiments (Fig. S5). The intact osteoblasts demonstrated a healthy response against the substrate collagen orientation; the cytoskeletal arrangement along the collagen fibers induced whole cell alignment in a preferred direction. A relationship between osteoblast alignment and the architecture of cell-produced bone matrix was proposed some decades ago^32^. Interestingly, we found that the microstructure of the bone matrix produced by aligned osteoblasts showed a preferred orientation depending on the cellular direction^12^,^13^. In particular, by controlling the substrate collagen anisotropy, we found that the degree of apatite orientation is quantitatively correlated to the level of osteoblast arrangement^11^. In this study, it was revealed for the first time that the direct interaction of osteoblasts with cancer cells induced a disruption of the osteoblast arrangement in response to the molecular orientation of collagen.

Osteoblasts and cancer cells were clearly identified by immunostaining of cancer cell markers (Fig. 5(b–d)). Under the conditions of the control monoculture, osteoblasts preferentially elongated along the substrate collagen orientation (Fig. 5(a,e)). However, the cellular arrangement was significantly disrupted during direct coculture with osteolytic melanomas and breast cancer cells as well as osteoblastic prostate cancer cells (Fig. 5(i)). Because the total number of cultured cells was adjusted to be equal in the monoculture and the coculture, the interaction with normal osteoblasts must have had negligible effects on cellular arrangement, in spite of the difference in shape and size of the cancer cells and the somatic cells. The effects of interaction with healthy somatic cells on the activity of osteoblasts should be studied in more detail. These results indicated that the intercellular communication between cancer cells and osteoblasts regulated the downstream signaling pathway and caused abnormal cytoskeletal rearrangement.

Intercellular adhesion has been shown to play a key role in the development of tissue shapes and structures required for organ function^33^,^34^. During complex organogenesis, physically associated cell populations are required for appropriate morphogenesis^35^. For example, the tightening of hindgut during embryogenesis has been shown to depend on localized asymmetric JAK/STAT signaling pathway activation^36^. In addition, cellular adhesion structures experience mechanical stress, as intercellular forces are prominent mediators regulating morphogenetic processes to maintain the physical integrity of tissues^37^. In metastasis, physical contact between cancer cells and endothelium has been shown to modulate endothelium phenotypes. As an intercellular communication highway, nanoscale intercellular membrane bridges have been shown to transport materials from metastatic cancer cells to the endothelium, which leads to transformation of the healthy endothelium into a pathological state^38^. Although the precise molecular mechanisms behind the cancer cell-mediated disruption of osteoblast alignment observed in this study are still unclear, the intercellular communication between bone and cancer cells, initiated by metastasized cancer cells, might cause impaired cytoskeletal dynamics in osteoblasts. Indeed, connexin 43 was highly expressed in isolated cancer cells and osteoblasts, indicating that physical contact via gap junctions on osteoblast arrangement is important, regardless of the type of cancer cell involved (Fig. 6).

Considering the complicated events involved in bone metastasis, a 3D coculture system would be necessary; previous studies that employed 3D coculture showed a significant impact on the cellular and molecular mechanisms underlying bone metastasis^16^,^17^,^18^,^19^,^20^. Further research involving 3D coculture is necessary to elucidate the molecular mechanisms regulating osteoblast arrangement. The establishment of 3D oriented collagen substrates that would enable the analysis of cellular anisotropic responses is now in progress.

**Figure 7:**
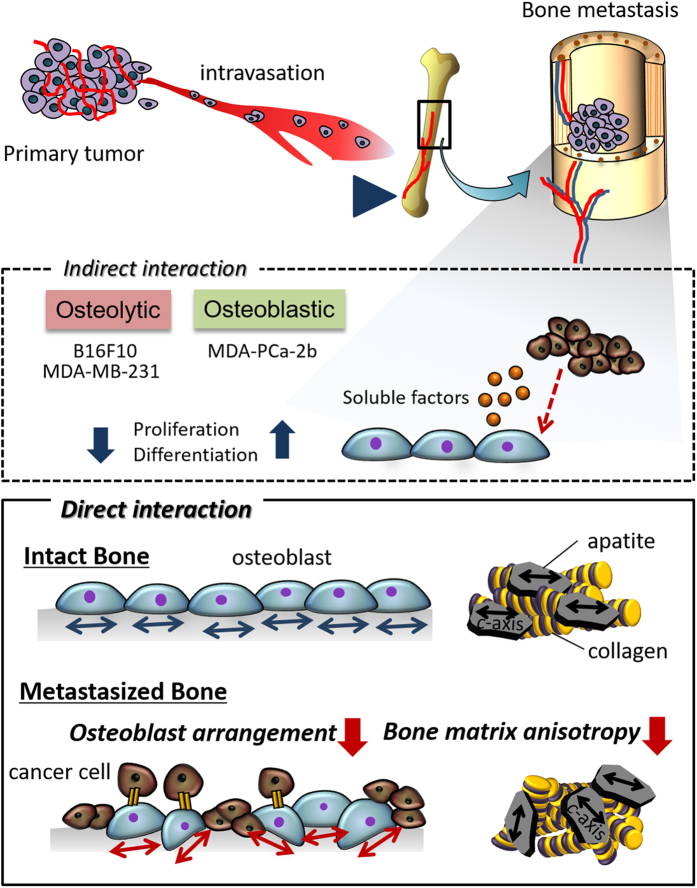
A schematic illustration of the effect of the interaction between cancer cells and osteoblasts on osteoblast function. The proliferation and differentiation behaviors of osteoblasts are regulated via soluble factors derived from cancer cells depending on the type of metastasis (osteolytic and osteoblatic). However, osteoblast arrangement, which determines the bone matrix microstructure is disrupted by direct interactions with cancer cells.

In conclusion, the present findings provide new insights into bone metastasis both for clinical applications and for understanding the underlying biological mechanism. The classical understanding of bone metastasis is limited to alteration in bone mass by metastatic cancer cells. However, we demonstrate that the extremely disrupted mechanical function in metastasized bone is derived from disrupted microstructure with less-aligned collagen/apatite matrix architecture^4^,^5^. In this study, the contribution of metastatic cancer cells to osteoblast arrangement was revealed for the first time. The osteoblasts failed to align under direct attack by cancer cells, whereas the cellular arrangement was not affected by the soluble factors secreted by cancer cells. The obtained findings indicate that the disrupted arrangement of osteoblasts induced by the physical contact with cancer cells facilitates the disorganized microstructure of metastasized bone (Fig. 7). The intercellular communication between bone and cancer can be a new therapeutic target for the recovery of anisotropic bone structure required for appropriate mechanical and biological function.

## Methods

### Osteoblast isolation and culture

Primary osteoblasts were isolated from the calvariae of neonatal mice^39^. Briefly, calvariae from postnatal 2–3 day old C57BL/6 mice were excised under aseptic conditions and placed in ice-cold α-modified Eagle’s Medium (α-MEM; GIBCO). The fibrous tissues around the bone were then gently removed. The calvariae were then subjected to a series of collagenase/trypsin (collagenase: Wako, Osaka, Japan; trypsin: nacalai tesque) digests at 37 °C for 15 min each. The first two digests were discarded, and supernatants 3, 4, and 5 were neutralized with α-MEM, pooled, and filtered using a 100-μm mesh (BD Biosciences, San Jose, CA). The filtrates were centrifuged and the resulting pellets were resuspended in α-MEM containing 10% fetal bovine serum (FBS) for cell culture. Cells were then diluted to 2.0 × 10^4^ cells/ml and seeded onto the fabricated specimens. The medium was changed twice a week. All cells were cultured in a humidified 37 °C incubator (Series 5400, NAPCO) containing 5% CO_2_ and 95% air.

All animal experiments were approved by the Osaka University Committee for Animal Experimentation (approval number: 22-2-0). All experiments were performed in accordance with the related guidelines and regulations for scientific and ethical animal experimentation.

### Culture of cancer cells

All cancer cells were purchased from American Type Culture Collection (ATCC, Manassas, VA). B16F10 and MDA-MB-231 cells were maintained in D-MEM (GIBCO) containing 10% FBS, 100 U/ml penicillin, and 100 μg/ml streptomycin. MDA-PCa-2b cells were maintained in ham’s F-12K (Wako) containing 20% FBS, 25 ng/ml cholera toxin, 10 ng/ml mouse epidermal growth factor, 0.005 mM phosphoethanolamine, 100 pg/ml hydrocortisone, 45nM selenious acid, and 0.005 mg/ml bovine insulin (Sigma). All cell culture experiments were performed in accordance with the protocols provided in cell line data sheets.

### Direct and indirect coculture of cancer cells and osteoblasts

For indirect coculture, cancer cell-derived conditioned media were collected as follows. The media from approximately 90% confluent cancer cells was replaced with serum free α-MEM. After 24 h, the obtained conditioned media was removed, centrifuged at 3000 rpm for 10 min, pooled, aliquoted, and frozen at −20 °C until use. Before using, 10% FBS, 100 U/ml penicillin, and 100 *μ*g/ml streptomycin were added to generate 100% concentration conditioned media. The obtained media was diluted with the same amount of α-MEM containing 10% FBS, 100 U/ml penicillin, and 100 *μ*g/ml streptomycin to generate 50% concentration conditioned media.

For direct coculture, the total cell number was adjusted such that it was equal in the control osteoblast monoculture and osteoblast-cancer cell coculture. The cell ratio of osteoblasts and cancer cells was determined by immunocytochemical analysis (Fig. S3). The criterion for determining the ratio of seeding cells was that the osteoblasts have at least one neighboring cancer cell. The seeding ratio of cells was estimated to be as follows: osteoblasts/B16F10 (10:1), osteoblasts/MDA-MB-231 (1:1), and osteoblasts/MDA-PCa-2b (1:2).

### Fabrication of oriented collagen substrates

Pepsin solubilized porcine skin collagen type-I (Nippi, Japan) was prepared at a concentration of 10 mg/ml in 0.02 N acetic acid. Oriented collagen substrates were produced using a hydrodynamic extraction method^40^. The process of collagen solution deposition into PBS was controlled by a three-axis robotic arm, which could regulate the direction of the collagen molecule fibrils.

### Cell proliferation assay

Primary osteoblasts were seeded at 2.0 × 10^4^ cells/ml and the media was then replaced with cancer cell-conditioned media or control media. After 72 h culture, cells were trypsinized and visible cells were counted with a hemocytometer by trypan blue dye exclusion. Giemsa staining was performed for the visualization of cells. The cells were fixed with methanol and stained with a 5% Giemsa aqueous solution. Images were obtained using a fluorescence microscope (Biozero, Keyence, Osaka, Japan).

### Cell viability assay

Live or dead cells were detected by immunoflourescence of Calcein-AM and Propidium iodide (PI) stained cells in accordance with the manufacture’s instructions (Double staining kit, Dojindo). Osteoblasts were seeded at 2.0 × 10^4^ cells/ml, and the media was then replaced with cancer cell-conditioned media or control media. After 72 h culture, cells were washed with PBS and then incubated with the double staining kit for 5 mins. The solution was replaced with PBS and all specimens were analyzed under a fluorescence microscope (Biozero, Keyence, Osaka, Japan). The number of living cells was counted and normalized relative to total cell number.

### Gene expression

Total RNA was extracted using TRIzol reagent (Invitrogen, Paisley, UK). We assessed the levels of ALP mRNA relative to GAPDH in osteoblasts by quantitative real-time PCR in accordance with the manufacture’s guidelines (Step-one, Applied Biosystems). The threshold number of cycles (Ct) was set within the exponential phase of the PCR reaction, and the ΔCt value for each target gene was calculated by subtracting the Ct value of GAPDH (internal control) from the target gene. Results are displayed as the relative expression (percentage) compared to GAPDH expression, and normalized relative to control cells.

### Immunocytochemistry

Cells were fixed in 4% paraformaldehyde and incubated in PBS-0.05% Triton X-100 (PBST) containing 1% normal goat serum (NGS; Invitrogen) for 30 minutes to block non-specific antibody binding sites. The cells were then incubated with mouse monoclonal antibodies against vinculin (Sigma-Aldrich) at 4 °C for 12 h. This step was followed by incubation with Alexa Fluor 546 conjugated anti mouse IgG (Molecular Probes, Invitrogen), Hoechst (invitrogen), and Alexa Fluor 488 conjugated phalloidin (Molecular Probes, Invitrogen). Finally, the cells were washed with PBST and mounted using the Prolong Gold antifade reagent (Molecular Probes, Invitrogen). Fluorescent images were observed using a fluorescence microscope, and processed using the Adobe Photoshop 10.0 software.

To identify the cancer cells, the following antibodies were applied as cancer cell markers; mouse anti MelanA (Santa Cruz) for B16F10, mouse anti CathepsinD (Abcam) for MDA-MB-231, and mouse anti PSA (Abcam) for MDA-PCa-2b. Intercellular adhesion was visualized using the following antibodies: rabbit-anti Connexin 43 (Cell Signaling) and rabbit anti-Cadherin 11 (Cell Signaling).

### Cell orientation

The orientation of the cells on the substrates was examined relative to the collagen molecular orientation by taking photographs of the fluorescent phalloidin staining images of the cells using a fluorescence microscope (Biozero, Keyence, Osaka, Japan). Cell orientation was quantitatively analyzed using the Cell Profiler software (Broad Institute, Cambridge, MA) (Fig. S4). The degree of cell orientation was characterized by the orientation order parameter for the 2-dimensional system, *f*_*2D*_, using the following equations^41^:


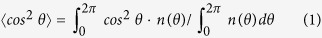



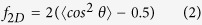


The degree of cell alignment *f*_2*D*_ takes a value ranging from −1 (cells perfectly aligned perpendicular to the collagen orientation), 0 (cells oriented randomly), to 1 (cells perfectly aligned parallel to the collagen orientation).

### Substrate collagen orientation

The degree of collagen orientation was analyzed with an optical microscope (Olympus, Tokyo, Japan) equipped with a birefringence measurement system (WPA-micro, Photonic Lattice, Miyagi, Japan). After culturing the cancer cells for 72 h, the cells on the substrate were washed with PBS and fixed with methanol for 10 min. After drying in air, the cells were stained with 5% Giemsa staining solution (Wako, Osaka, Japan) for 10 min. Cells were then washed in double-distilled water (DDW) and air-dried. The collagen substrate without the cancer cells were treated by the same procedure described above and then used as the control group. The specimens were imaged with a 50-× objective lens. The polarization axis with the greater index of refraction (slow axis) was detected using the integrated polarizer. The average orientation of the slow axis for each pixel was analyzed using the WPA-VIEW software (version 2.4.2.9, Pho-tonic Lattice).

### Statistical analysis

Statistical significance was assessed by one-way ANOVA, followed by Tukey’s *post hoc* test. A significance of *P* < 0.05 was required for rejection of the null hypothesis.

## Additional Information

**How to cite this article:** Kimura, Y. *et al*. Alteration of osteoblast arrangement via direct attack by cancer cells: New insights into bone metastasis. *Sci. Rep.* **7**, 44824; doi: 10.1038/srep44824 (2017).

**Publisher's note:** Springer Nature remains neutral with regard to jurisdictional claims in published maps and institutional affiliations.

## Supplementary Material

Supplementary Information

## Figures and Tables

**Figure 1:**
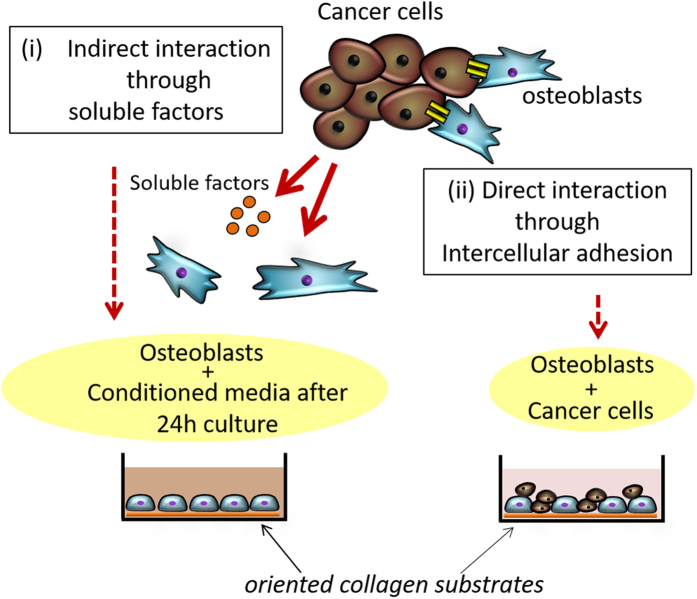
A schematic illustration of (i) indirect and (ii) direct coculture systems of osteoblasts and cancer cells. Primary osteoblasts were cultured on oriented collagen substrate under indirect coclture (cancer cell-derived conditioned media) and direct coculture with cancer cells.
