# Supplementary material for: Alteration of osteoblast arrangement via direct attack by cancer cells: New insights into bone metastasis
Source: Sci Rep. 2017 Mar 17;7:44824. doi: 10.1038/srep44824 (PMC5356003; doi:10.1038/srep44824)
Supplement: Supplementary Information [file srep44824-s1.pdf]

SUPPLEMENTARY INFORMATION
For

Alteration of osteoblast arrangement via direct attack by cancer cells: New insights into bone metastasis

Yumi Kimura, Aira Matsugaki, Aiko Sekita and Takayoshi Nakano*

Division of Materials and Manufacturing Science, Graduate School of Engineering, Osaka University, 2-1 Yamadaoka, Suita, Osaka 565-0871, Japan

* Corresponding author: nakano@mat.eng.osaka-u.ac.jp

Supplementary Figure S1

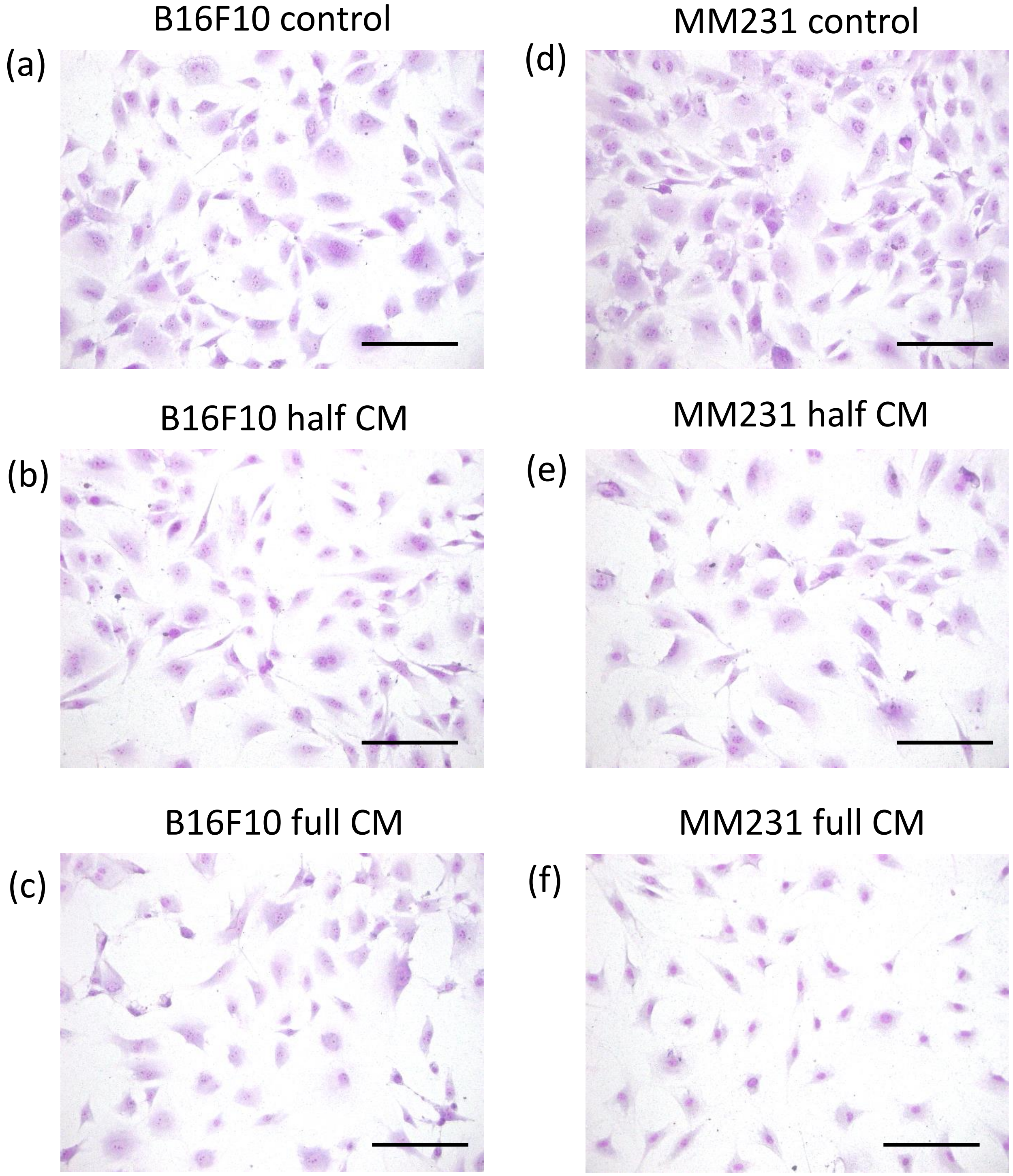

Supplementary Figure S1

Giemsa staining of osteoblasts cultured with cancer cell-conditioned media ((a), (d): control, (b), (e): 50% concentration and (c), (f): 100% concentration of (a), (b), (c): mouse melanoma B16F10, (d), (e), (f): human breast cancer MDA-MB-231 cells conditioned media). Scale bar: 100 μm.

Supplementary Figure S2

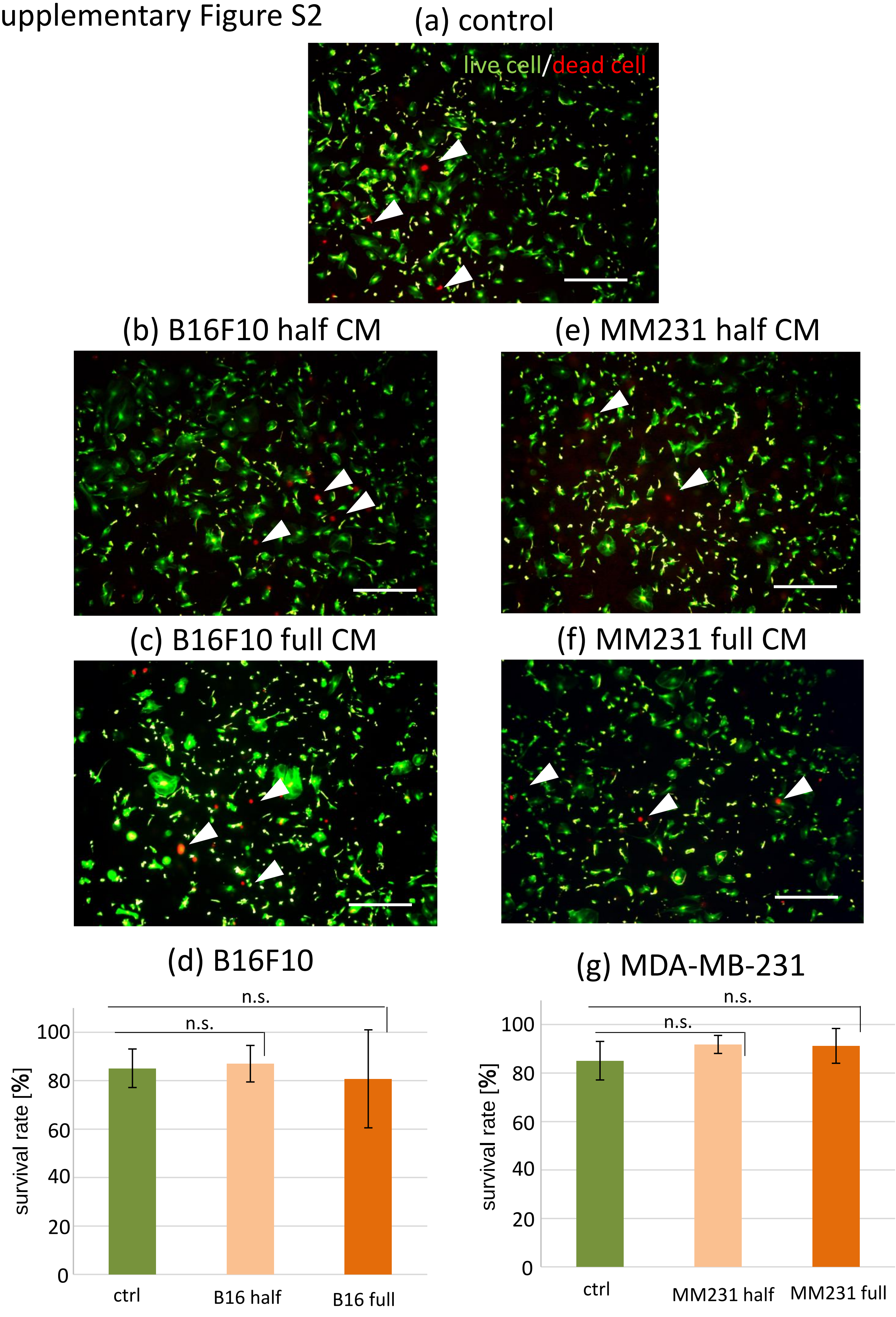

Supplementary Figure S2

Cell viability analysis of osteoblasts cultured with cancer cell-conditioned media ((a): control, (b), (e): 50% concentration and (c), (f): 100% concentration of (b), (c): mouse melanoma B16F10, (e), (f): human breast cancer MDA-MB-231 cells conditioned media). Green: live cells, Red: dead cells. Scale bar: 500 μm. The survival rate of osteoblasts was not significantly different between cells cultured with and without (d) B16F10 or (g) MDA-MB-231conditioned media.

Supplementary Figure S3

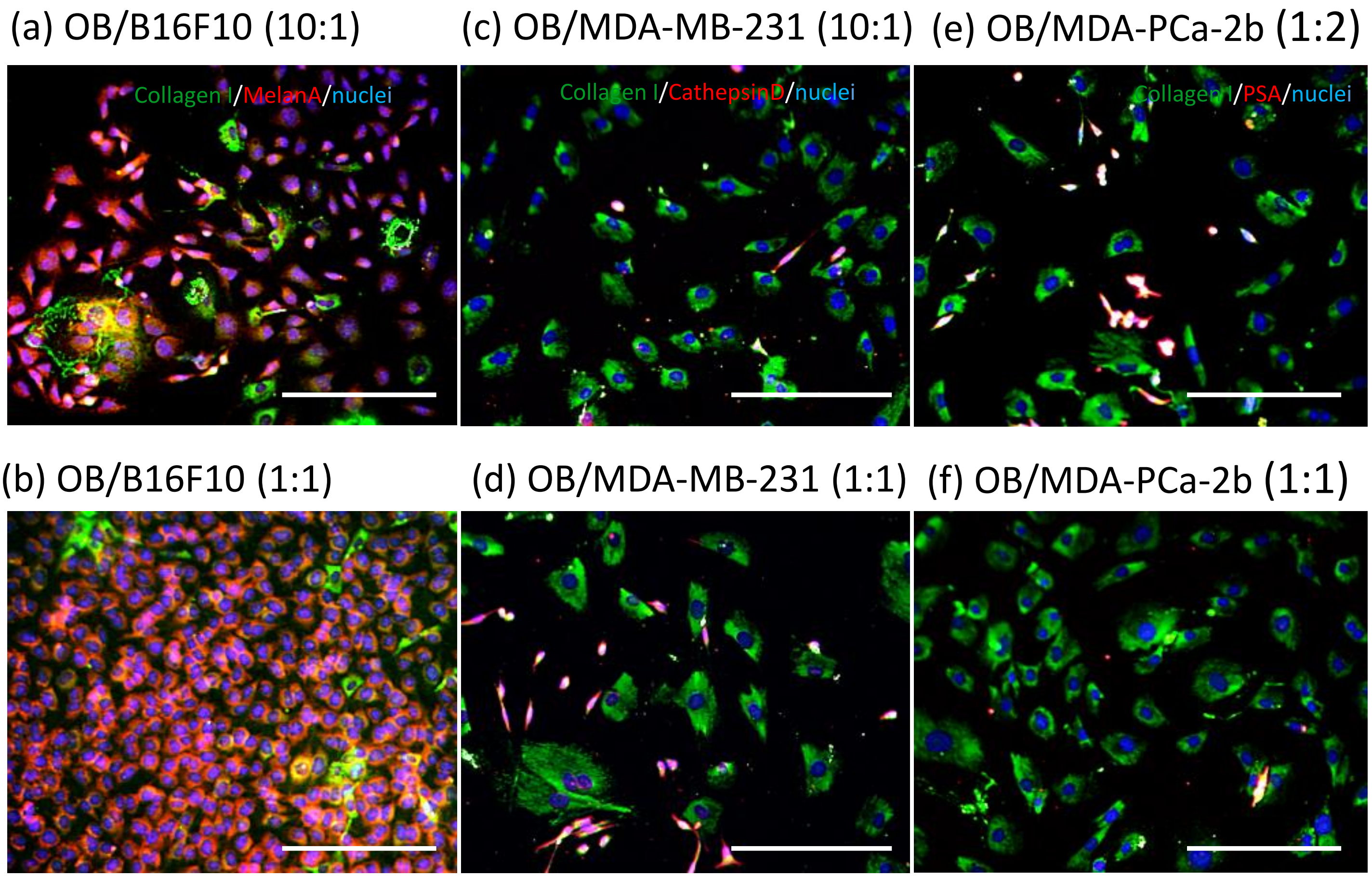

Supplementary Figure S3

Immunocytochemical analysis of the seeding ratio of osteoblasts and cancer cells under direct coculture. Osteoblasts and cancer cells were seeded at a cell ratio of osteoblasts:B16F10- (a) 10:1, (b)1:1, Osteoblast:MDA-MB-231- (c) 10:1, (d) 1:1, Osteoblast:MDA-PCa-2b- (e)1:2, (f) 1:1. (a), (b) Green: Collagen type I, red: Melan A, blue: nuclei. (c), (d) Green: Collagen type I, red: Cathepsin D, blue: nuclei. (e), (f) Green: Collagen type I, red: PSA, blue: nuclei. Scale bar: 100 μm.

Supplementary Figure S4

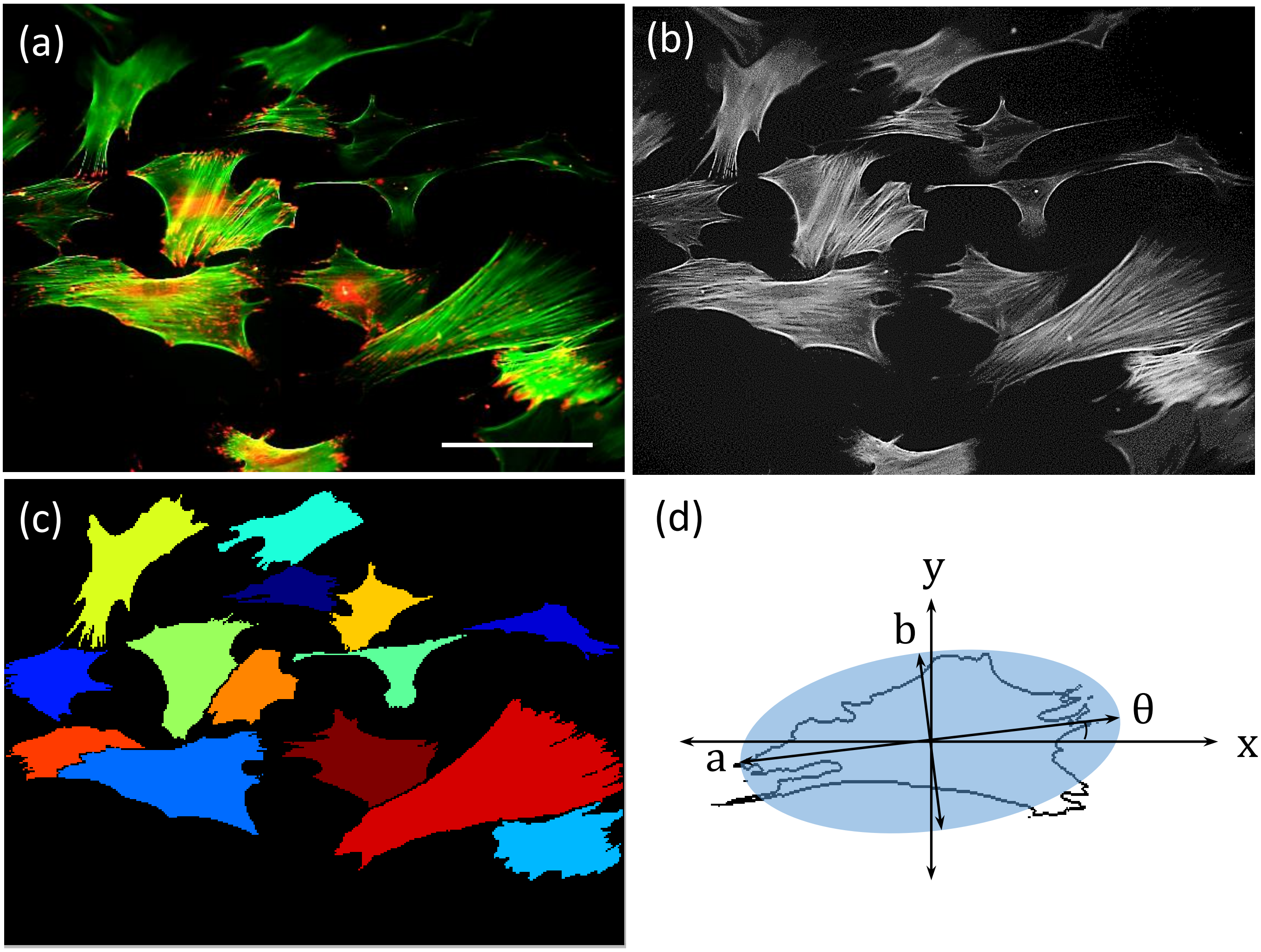

Supplementary Figure S4

Morphological analysis of cells cultured on oriented collagen substrates. An immunocytochemical image of an osteoblast (Green: F-actin, red: vinculin, blue: nuclei) (a) was binarized (b), and outlined cell shape images were obtained using the Cell Profiler software (ver. 2.0, http://cellprofiler.org/). (c). Individual cells are identified in different colors. An elliptical approximation was applied. The major: a, and the minor axes of cell shape: b, were determined. The cellular angle was also determined θ (d). Scale bar: 100 μm.

Supplementary Figure S5

(a) control

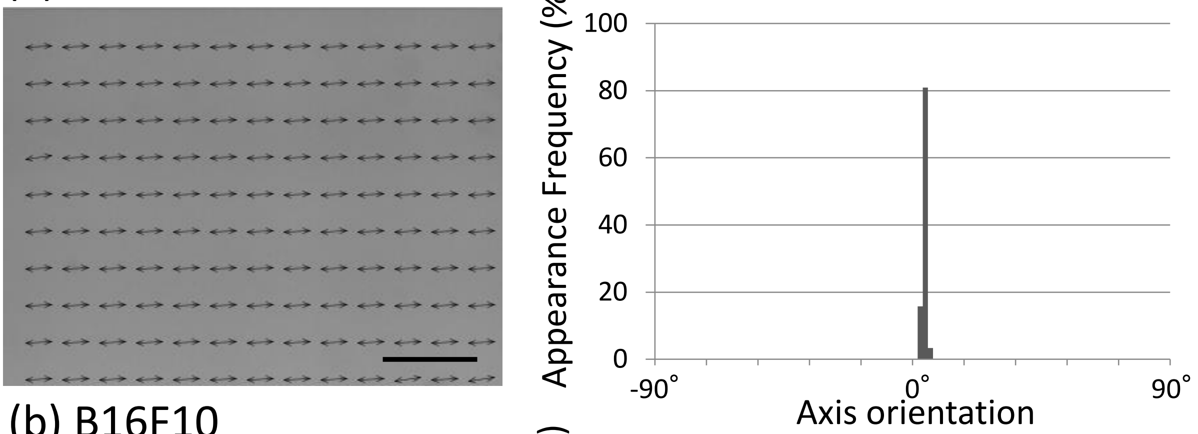

(b) B16F10

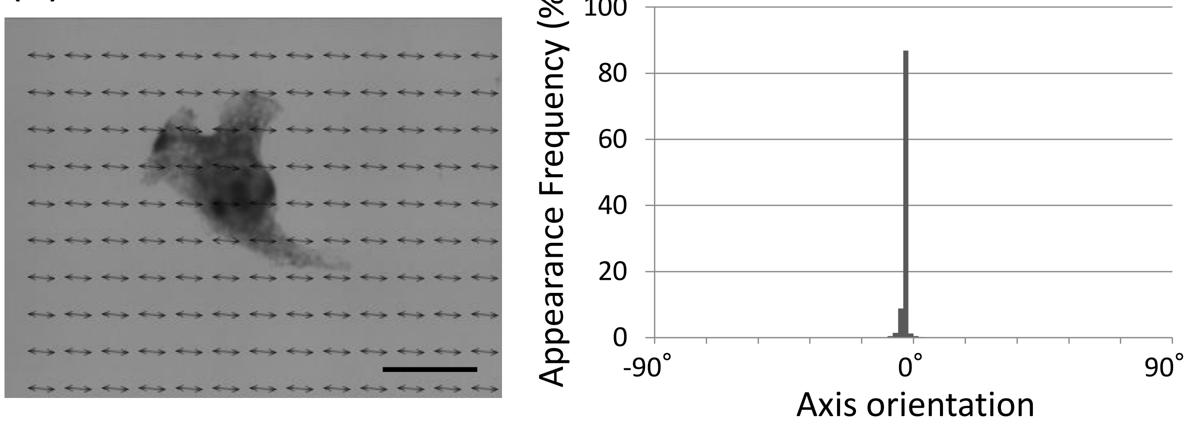

(c) MDA-MB-231

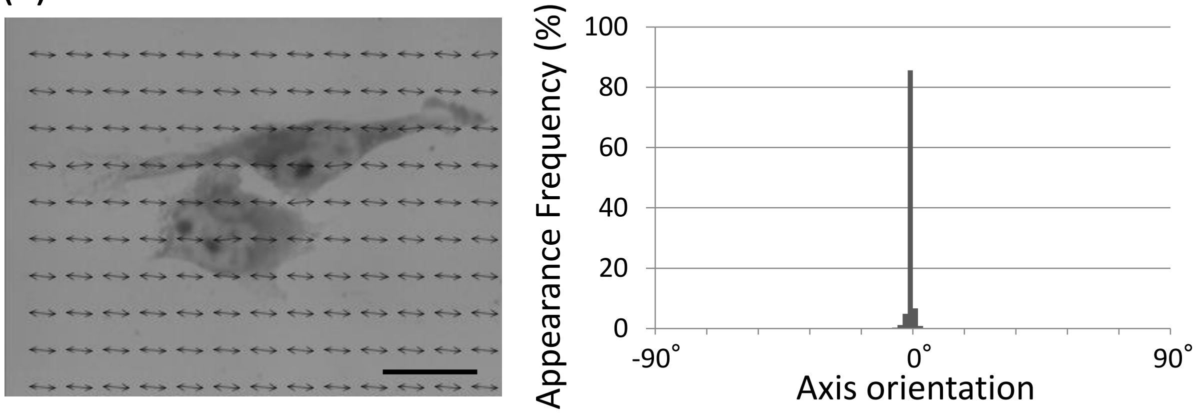

(d) MDA-PCa-2b

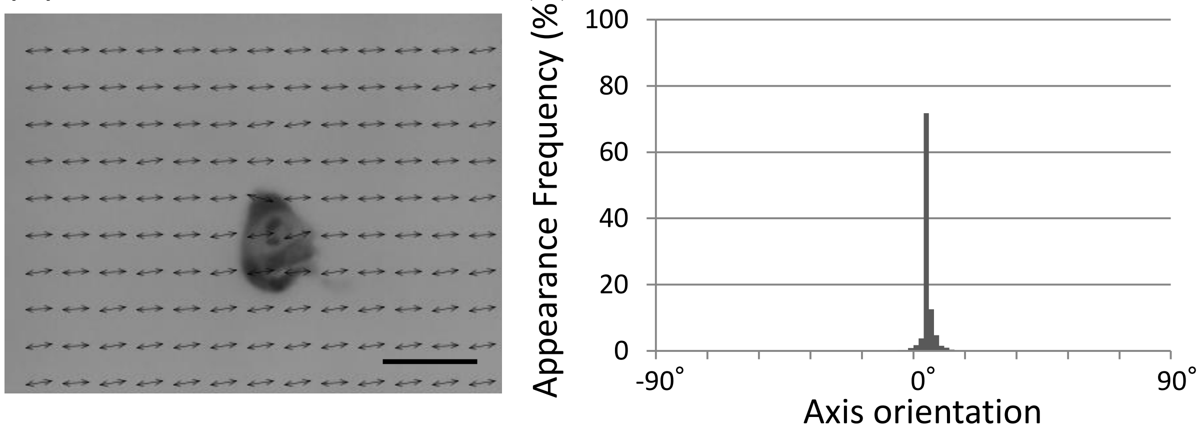

Supplementary Figure S5

Birefringence analysis of oriented collagen substrate (a) without cancer cells and with cancer cells, including (b) mouse melanoma B16F10, (c) human breast cancer MDA-MB-231, and (d) human prostate cancer MDA-PCa-2b. Left column: optical microscopic images of the cells. Arrows represent the substrate collagen orientation, which were obtained using the WPA-VIEW software (version 2.4.2.9, Photonic Lattice, https://www.photonic-lattice.com/ja/downloads/domo/). Right column: Distribution of collagen orientation. Scale bar: 20 µm.
